# A novel action of follicle-stimulating hormone in the ovary promotes estradiol production without inducing excessive follicular growth before puberty

**DOI:** 10.1038/srep46222

**Published:** 2017-04-11

**Authors:** Charlotte M. François, Florence Petit, Frank Giton, Alain Gougeon, Célia Ravel, Solange Magre, Joëlle Cohen-Tannoudji, Céline J. Guigon

**Affiliations:** 1 Sorbonne Paris Cité, Université Paris-Diderot, CNRS, INSERM, Biologie Fonctionnelle et Adaptative UMR 8251, Physiologie de l’Axe Gonadotrope U1133, Paris, France; 2 APHP CIB GHU Sud Henri Mondor, INSERM IMRB U955, Eq.07, Faculté de Médecine, Creteil, France; 3 Inserm U1052, CRCL, Faculté de Médecine Laennec, Lyon, France; 4 CHU de Rennes - Université de Rennes 1, Laboratoire de Biologie de la Reproduction, INSERM U1085-IRSET, France

## Abstract

In cyclic females, FSH stimulates ovarian estradiol (E2) production and follicular growth up to the terminal stage. A transient elevation in circulating FSH and E2 levels occurs shortly after birth. But what could be the action of FSH on the ovary during this period, and in particular how it stimulates ovarian steroidogenesis without supporting terminal follicular maturation is intriguing. By experimentally manipulating FSH levels, we demonstrate in mice that the mid-infantile elevation in FSH is mandatory for E2 production by the immature ovary, but that it does not stimulate follicle growth. Importantly, FSH increases aromatase expression to stimulate E2 synthesis, however it becomes unable to induce cyclin D2, a major driver of granulosa cell proliferation. Besides, although FSH prematurely induces luteinizing hormone (LH) receptor expression in granulosa cells, LH pathway is not functional in these cells to induce their terminal differentiation. In line with these results, supplying infantile mice with a superovulation regimen exacerbates E2 production, but it does not stimulate the growth of follicles and it does not induce ovulation. Overall, our findings unveil a regulation whereby high postnatal FSH concentrations ensure the supply of E2 required for programming adult reproductive function without inducing follicular maturation before puberty.

Postnatal gonadotrope axis activation has been poorly documented in mammal species. This period of transient elevation in circulating gonadotropins, *i.e.*, luteinizing hormone (LH) and follicle-stimulating hormone (FSH) occurs in the first weeks (rodents, cattle) or months (chimpanzees, humans) after birth in both males and females. It follows a sex dimorphic pattern, with circulating FSH being more elevated than LH in females, while it is the opposite in males^1^,^2^,^3^,^4^,^5^,^6^,^7^. It is accompanied by sex-specific profiles of sex steroids (testosterone in males and estradiol [E2] in females) that contribute to postnatal male and female sexual development^8^,^9^,^10^,^11^,^12^. Gonadotropin and sex steroid levels become markedly elevated, and therefore this period has been named ‘mini-puberty of infancy’ in humans^5^,^9^. It is followed by a period of relative quiescence of pituitary gonadotrope and gonadal activities, and then by their re-activation shortly before puberty.

In cyclic females, FSH plays an important role in supporting follicular development up to the terminal stage of maturation^13^. A number of studies have documented the similarities of FSH actions in the ovary of sexually immature and mature females. Indeed, as in cyclic females, FSH acts *via* its receptor (FSHR) exclusively located on granulosa cells to convert thecal-derived androgens into estrogens in prepubertal females^14^,^15^,^16^. Besides, FSH also regulates follicular growth during this period as shown in infantile rodents by the arrest of folliculogenesis at the preantral/early antral stage in *Fshr*^−/−^ and *Fshb*^*−/−*^ mice and by its restoration induced by an FSHR agonist in hypophysectomized rats^17^,^18^,^19^. FSH regulation of early follicular growth in infantile mice has also been demonstrated *in vitro*^20^. On the other hand, despite the important elevation in circulating FSH in prepubertal females and the associated production of E2, follicles do not grow beyond the antral stage and no ovulation occurs^21^,^22^,^23^,^24^,^25^. These latter observations are intriguing given the well-documented stimulatory action of FSH on the growth of follicles^13^. Therefore, during this period, FSH action on the ovary cannot simply be extrapolated from our knowledge on adult physiology. Hence, in-depths studies are required to thoroughly analyze gonadotrope axis activation and to establish the effect of elevated FSH concentrations on the function of the immature ovary.

Here we used the mouse as an experimental model to determine how elevated postnatal FSH levels regulate key ovarian physiological processes, *i.e.*, steroidogenesis, follicular growth and maturation during the infantile period. We used a pharmacological approach to suppress FSH specifically at its peak during mid-infantile life. The current study clearly shows that the marked elevation in FSH levels occurring during the postnatal period is mandatory for the significant production of E2 by the immature ovary. However, it does not stimulate preantral/early antral follicle growth and it does not stimulate terminal follicular maturation. Our studies provide some molecular cues to explain this unprecedented uncoupling in FSH-mediated regulation of ovarian steroidogenesis and follicular growth during the prepubertal period.

## Results

### Postnatal FSH levels and ovarian steroidogenesis are dramatically elevated during the infantile period but follicles do not grow beyond the early antral stage

**Figure 1:**
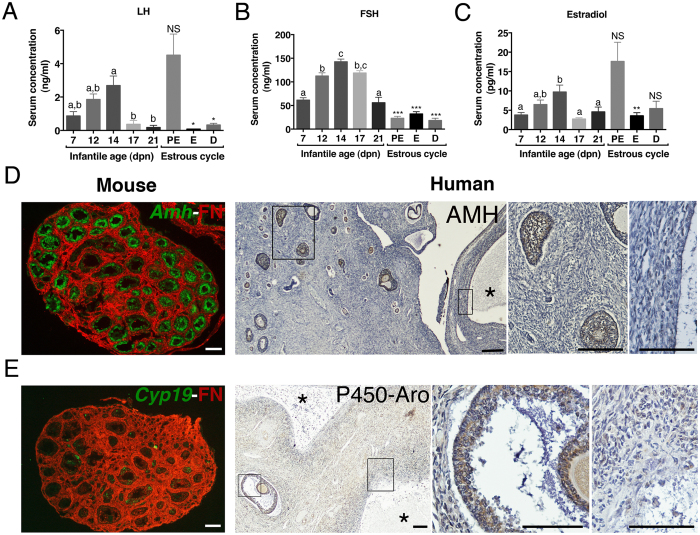
High activity of the gonadotrope axis during the postnatal period. (**A,B**) Serum levels of LH (**A**) and FSH (**B**) between 7–21 dpn and in cyclic mice at proestrus (PE), estrus (**E**) and diestrus 2 (**D**). LH and FSH levels were simultaneously assayed by Luminex assay in serum samples from 5 to 15 females/group. (**C**) Circulating levels of estradiol between 7–21 dpn and in cycling females at proestrus (PE), estrus (**E**) and diestrus 2 (**D**) as determined by GC-MS in 4–6 pooled (postnatal) or 3–6 (adult) mouse serum. Error bars indicate SEM. Different letters indicate significant differences (*P* < 0.05) between ages during the postnatal period, as analyzed by one-way ANOVA. Statistical difference by Student-*t* test with *P < 0.05, **P < 0.01, ***P < 0.001 between 14 dpn females and a given stage of the estrous cycle. NS, not significant. (**D,E**) Assessment of folliculogenesis and steroidogenesis in a 14-dpn mouse ovary by *in situ* hybridization of *Amh* or *Cyp19a1* mRNAs (purple color digitally converted into green), respectively, and immunodetection of fibronectin (FN, in red) on the same ovarian section. (**E**) Immunohistochemistry for AMH or aromatase (brown staining) in a human ovary at 2 months counterstained by hematoxylin (blue color). Stars (*) show large follicular cysts. Rectangles within left panels are enlarged to show AMH- or aromatase-positive preantral/antral follicles (middle panels) and negative follicular cysts (right panels). Scale bars: 100 μm.

Since postnatal gonadotrope axis activation has been poorly documented in the mouse, we first analyzed the serum profiles of LH, FSH and E2 during the infantile period from 7 to 21 days postnatal (dpn). To better evaluate the magnitude of pituitary and ovary activity during this period, we also monitored these endocrine parameters in adult females at the time of ovulation (proestrus, PE) and at other stages of the estrous cycle. Both LH and FSH levels were elevated in immature females but FSH was largely predominant (Fig. 1A and *B*). Circulating LH barely reached LH preovulatory surge values even at its peak at 14 dpn, and it declined sharply afterwards (Fig. 1A). By contrast, circulating FSH was ~5 fold higher than in adults at 14 dpn, and it decreased after 17 dpn (Fig. 1B). Ovarian LH and FSH receptivity, as assessed by *Lhcgr* and *Fshr* transcript abundance, increased rapidly during this period (Supplementary Fig. 1), further emphasizing the fact that growing follicles (GFs) become sensitive to gonadotropins early after birth in rodents^15^,^16^. By using the gold standard method for steroid measurement, *i.e.*, gas chromatography coupled with mass spectrometry (GC-MS), we found that E2 production increased concomitantly to the rise in gonadotropin levels to attain a peak at 14 dpn (Fig. 1C). We further observed an overall increase in the relative abundance of major components of the sex steroid biosynthesis pathway (Supplementary Fig. 1).

At the time of postnatal FSH and LH elevation, numerous GFs up to the preantral/early antral stage with a diameter of ~120–150 μm (mean surface of follicles: ~6 × 10^3^ μm^2^) were present in the central part of the murine ovary (data not shown), as reported^26^. They correspond to the first follicular wave of follicles developing immediately after their formation^27^. They expressed AMH (anti-Müllerian hormone), used as a marker of healthy GFs (Fig. 1D). We observed a somehow similar pattern of follicular growth in ovaries from human infants at an equivalent period of development (2–6 months old; n = 4 ovaries) with numerous AMH positive GFs up to the antral follicle stage (≤2 mm in diameter) (shown at 2 months in Fig. 1D). Those GFs in both mouse and human ovaries also expressed the enzyme that is responsible for the conversion of androgens into estrogens, *i.e.*, aromatase (Fig. 1E), which normally appears in granulosa cells from late antral/preovulatory follicles in sexually mature females (data not shown), as reported^28^. In addition, human infant ovaries exhibited follicular cysts (9–10 mm in diameter) consisting of a large cavity surrounded by a thick layer of thecal cells that displayed neither AMH nor aromatase expression (Fig. 1E). Follicular cysts are observed throughout childhood in human ovaries^29^.

### Elevated postnatal FSH concentrations are necessary to promote E2 biosynthesis but not for follicular growth

**Figure 2:**
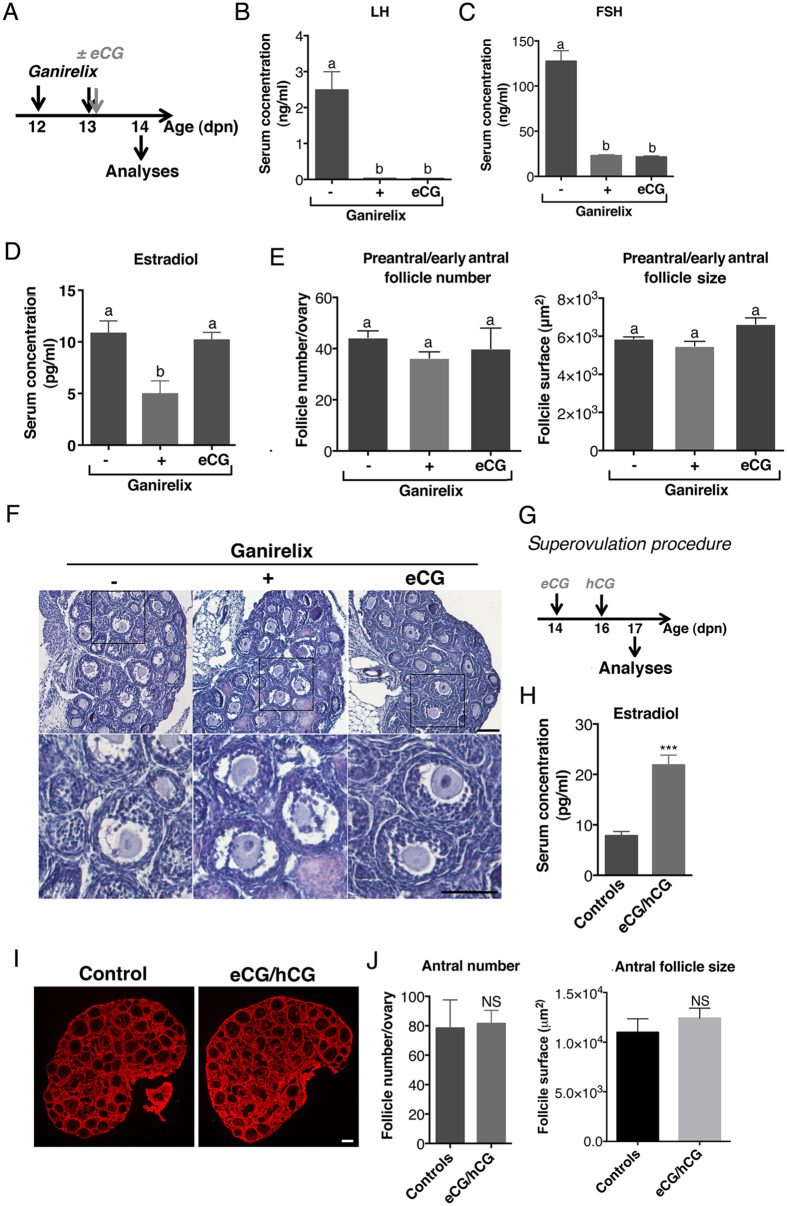
The high levels of gonadotropins stimulate E2 biosynthesis but have no effect on follicle growth during the infantile period. (**A**) Schematic representation of the procedure used to experimentally decrease FSH levels between 12 and 14 dpn in mice. (**B** and **C**) Circulating LH (**B**) and FSH (**C**) levels in females treated with saline (−), Ganirelix (+) or Ganirelix plus eCG (eCG). Gonadotropins were simultaneously assayed in the serum from 9 females/group. (**D**) Circulating E2 levels in 5–6 pooled serum in females treated with saline (−), Ganirelix (+) or Ganirelix plus eCG (eCG), as determined by GC-MS. (**E,F**) Morphometric analyses of preantral/early antral follicle number (left) and size (right) (**E**) performed on histological sections of hematoxylin/eosin stained ovarian sections (**F**) of females treated with saline (−), Ganirelix (+) or Ganirelix plus eCG (eCG) from 3–4 ovaries/group. (**G**) Schematic view of the superovulation procedure. (*H*) Effect of the superovulation treatment (eCG/hCG) on circulating E2 levels. (**I,J**) Effects of the superovulation treatment on follicular growth as shown by fibronectin immunodetection (red) to visualize follicle morphology (**I**) and morphometric analyses of antral follicle number and size (**J**) from at least 3 ovaries/group. In **F** and **I**, micrographs are representative of the observations performed on 3 ovaries/group. Scale bars: 100 μm. In **E** and **J**, morphometric analyses were performed by ImageJ, as described in *Materials and Methods*.

To determine the role of the postnatal elevation in FSH concentrations on both E2 production and follicular growth, female mice were treated by the GnRH receptor antagonist Ganirelix to decrease the FSH peak (12–14 dpn), and some of them were supplemented with the exogenous gonadotropin eCG to substantially re-activate FSH signaling pathway (Fig. 2A). Ganirelix treatment suppressed LH levels and induced an 80% reduction in FSH levels (Fig. 2B and C). It was accompanied by a 2-fold decrease in circulating E2 levels, but these levels remained normal after co-treatment with eCG (Fig. 2D). Notably, in spite of these dramatic effects on ovarian steroidogenesis caused by reduced FSH action, the growth of preantral/early antral follicles of the first wave remained unaltered as shown by their normal number and size (Fig. 2E and F). As a complementary experiment, we administered a superovulation regimen consisting of eCG at 14 dpn for boosting follicular growth and then of hCG at 16 dpn for inducing ovulation through activation of LH signaling. Animals were sacrificed at 17 dpn to assess the effect of the treatment on ovarian steroidogenesis and follicular growth (Fig. 2G). Compared with 14 dpn ovaries, folliculogenesis had slightly progressed in 17 dpn control females, with antral follicles in the central part of the ovary exhibiting a diameter >200 μm (mean surface of follicles: ~1.1 × 10^4^ μm^2^). Interestingly, the treatment led to the increase by ~ two fold in circulating E2 levels as compared with those in control females (Fig. 2H). However, there was no alteration in follicular growth and no ovulation in mice under surperovulation regimen (Fig. 2I and J).

### The refractoriness of follicular growth to high FSH levels may result from the loss of FSH-mediated *Ccnd2* expression

**Figure 3:**
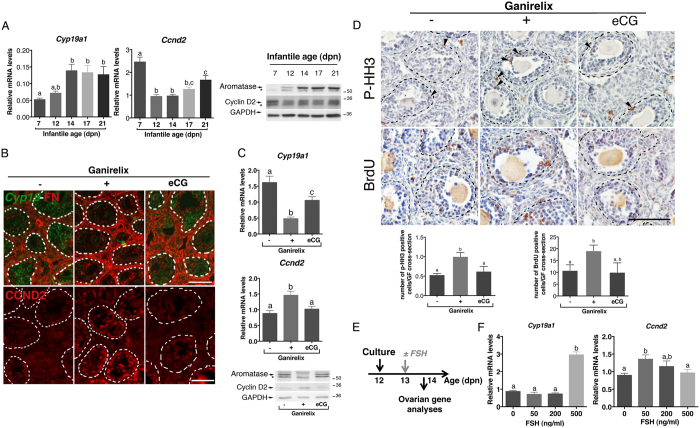
Distinct regulatory action of FSH on *Cyp19a1* and *Ccnd2* expression in infantile ovaries. (**A**) Relative intra-ovarian contents of both *Cyp19a1* and *Ccnd2* mRNAs and proteins (aromatase and cyclin D2, respectively) in 7–21 dpn mice. The relative abundance of mRNAs was determined by RT-qPCR and normalized to the mRNA levels of *Hprt* (5–7 ovaries/group). Aromatase and CCND2 protein abundance was analyzed by Western blots, with GAPDH as a loading control. Representative immunoblots are shown. Stars show non-specific bands. (**B**) Merged images of *in situ* hybridization assays showing *Cyp19a1* mRNAs (purple color digitally converted into green color), and immunolabeled fibronectin (FN, red) and immunofluorescence study of CCND2 in the ovaries of females treated with saline (−), Ganirelix (+) or Ganirelix plus eCG (eCG). Dashed lines represent the outlines of growing follicles located in the core of the ovary. (**C**) Relative intra-ovarian contents of both *Cyp19a1* and *Ccnd2* mRNAs and proteins (aromatase and cyclin D2, respectively) in females treated with saline (−), Ganirelix (+) or Ganirelix plus eCG (eCG). Data are the mean ± SEM. The relative abundance of mRNAs was determined by RT-qPCR after normalization to the levels of *Hprt* mRNAs (5–8 ovaries/group). Aromatase and CCND2 protein abundance was analyzed by Western blots with GAPDH as a loading control. Representative immunoblots are shown. (**D**) Ovarian immuno-detection of phosphorylated histone H3 (p-HH3) and of BrdU, and quantification of the mean number of p-HH3 positive granulosa cells per cross-sectioned preantral/early antral follicles of females treated with saline (−), Ganirelix (+) or Ganirelix plus eCG (eCG). Dashed lines represent outlines of follicles. Arrowheads show positive granulosa cells. (**E**) Schematic representation of the experimental set-up for ovarian cultures. (**F**) Relative intra-ovarian content of both *Cyp19a1* and *Ccnd2* mRNAs in cultured postnatal ovaries (3–13 ovaries/group) in response to different concentrations of purified FSH. Data were obtained by RT-qPCR and normalized to the mRNA levels of *Hprt*. In graphs of (**A**,**C**,**D** and **F**) data are the mean ± SEM. Data were analyzed by one-way ANOVA, with distinct letters indicating significant differences between ages or groups (*P* < 0.05). Scale bars: 100 μm.

In the ovary, FSH up-regulates the expression of aromatase (*Cyp19a1*) and cyclin D2 (*Ccnd2*), which are involved in E2 production and follicular growth, respectively^13^,^30^,^31^. CCND2 is specifically expressed in granulosa cells of GFs at the difference of the other D-type cyclins^30^,^31^. To understand how FSH stimulates steroidogenesis but not follicular growth in infantile ovaries, we analyzed its action on the expression of these two genes. Study of the ontogeny of both *Cyp19a1* and *Ccnd2* expression revealed that their relative abundance markedly varied during the infantile period (Fig. 3A). While *Cyp19a1* transcript and protein levels grossly followed the same pattern as circulating FSH levels, interestingly *Ccnd2* mRNA and protein levels displayed an opposite pattern (Figs 1B and 3A). To better understand the action of FSH on *Cyp19a1* and *Ccnd2* expression in immature ovaries, we examined the effect of Ganirelix treatment, with or without co-administration of eCG, on the regulation of these two genes. Ganirelix treatment significantly decreased *Cyp19a1* mRNA and protein expression in preantral/early antral follicles (Fig. 3B and C), in line with the observed reduction in E2 levels (Fig. 2D). However, it increased *Ccnd2* mRNA and protein levels in these follicles (Fig. 3B and C). Supplying eCG prevented the effects of the Ganirelix treatment on both *Cyp19a1* and *Ccnd2* expression (Fig. 3B and C).

To determine whether these alterations in FSH-regulated *Ccnd2* expression had an impact on granulosa cell proliferation in preantral/early antral follicles, we examined by immunohistochemistry the expression of a mitosis marker, *i.e.*, phosphorylated histone H3 (p-HH3)^32^ and the incorporation of BrdU under these experimental conditions (Fig. 3D). Importantly, Ganirelix treatment increased by ~2 fold the number of granulosa cells expressing p-HH3 or with BrdU incorporation as compared with controls (Fig. 3D), and this effect was prevented by co-administration of eCG (Fig. 3D). To gain further insights into this observed differential regulation of *Cyp19a1* and *Ccnd2* by FSH in immature ovaries, we analyzed the effect of increasing concentrations of FSH on their expression using an *in vitro* culture system (Fig. 3E). Ovaries from 12-dpn mice were cultured in serum-free medium, and then treated with different concentrations of purified FSH (50, 200 and 500 ng/ml). At the highest dose, FSH up-regulated the expression of *Cyp19a1*, but it did not alter that of *Ccnd2* (Fig. 3F). In contrast, at the lowest dose, FSH significantly augmented *Ccnd2* mRNA levels but not those of *Cyp19a1* mRNAs (Fig. 3F).

### Lack of FSH-induced terminal follicular maturation is associated with the inefficiency of LH signaling in granulosa cells

**Figure 4:**
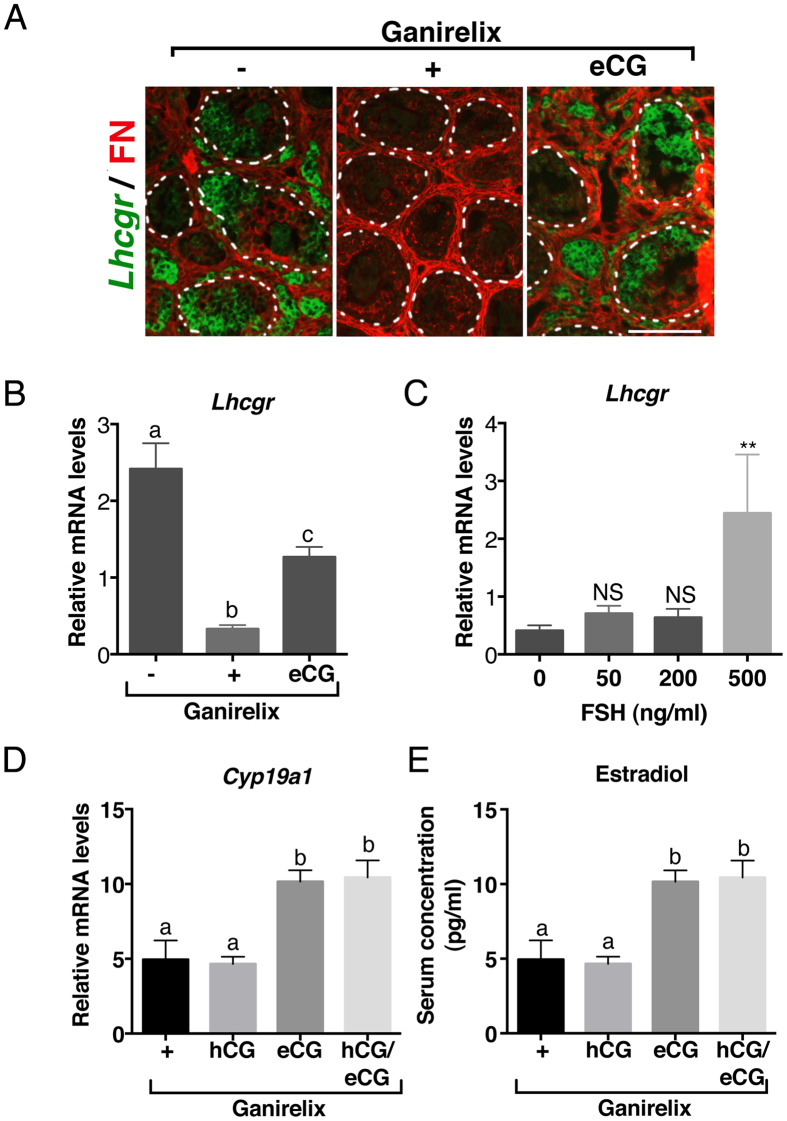
The elevated FSH levels of the infantile period stimulate the expression of LHCGR but probably not the activation of LHCGR signaling in granulosa cells. (**A**) Merged images of *in situ* hybridization assay detecting *Lhcgr* mRNAs (purple color digitally converted into green color) with immunolabelled fibronectin (FN, red) in preantral/early antral follicles of females treated with saline (−), Ganirelix (+) or Ganirelix plus eCG (eCG). (**B**) Relative intra-ovarian contents of *Lhcgr* mRNAs in the ovaries of females treated with saline (−), Ganirelix (+) or Ganirelix plus eCG (eCG) (8–9 ovaries/group). (**C**) Relative intra-ovarian contents of *Lhcgr* mRNAs in cultured postnatal ovaries (3–10 ovaries/group) in response to different concentrations of purified FSH. (*D*) Relative intra-ovarian contents of *Cyp19a1* mRNAs in the ovaries of females treated with Ganirelix (+), Ganirelix plus hCG (hCG), Ganirelix plus eCG (eCG), or Ganirelix plus hCG and eCG (hCG/eCG) (7–9 ovaries/group). (**E**) Circulating E2 levels in 5–6 pooled serum in females treated with Ganirelix (+), Ganirelix plus hCG (hCG), Ganirelix plus eCG (eCG), or Ganirelix plus hCG and eCG (hCG/eCG), as determined by GC-MS. Data in *B, D* and *E* were analyzed by one-way ANOVA, with distinct letters indicating significant differences between groups. Data in *C* were analyzed by Student t-test with **P < 0.01 in treated samples *versus* control group. Scale bars: 100 μm.

When GFs reach the terminal follicular stage in cyclic females, FSH induces LHCGR expression in addition to up-regulating *Cyp19a1* expression in granulosa cells; the preovulatory surge in LH then further increases E2 production and leads to the release of EGFR ligands to activate the ovulatory cascade^13^,^33^. The dramatic elevation in FSH levels together with the premature maturation of GFs demonstrated by *Cyp19a1* expression in preantral/early antral follicles of infantile ovaries led us to seek the effect of FSH on LHCGR expression and functionality during the infantile period. Strikingly, *Lhcgr* transcripts were not only observed in thecal cells of GFs as expected, but also in granulosa cells from antral follicles (Fig. 4A). *Lhcgr* expression in granulosa cells disappeared following Ganirelix treatment and was partially restored after eCG supply (Fig. 4A and B). This stimulation of *Lhcgr* expression by high FSH levels was further demonstrated *in vitro* in infantile ovaries wherein a high, but not a low, FSH concentration efficiently increased *Lhcgr* mRNA abundance (Fig. 4C). To assess whether LH signaling was functional in granulosa cells, we examined the effect of hCG on both *Cyp19a1* expression and circulating E2 levels in infantile Ganirelix-treated females supplemented or not with eCG. There was no induction in *Cyp19a1* expression and no increase in E2 levels in hCG-treated females (Fig. 4D and E). Besides, the expression of *Ccnd2*, which is negatively regulated by LH in granulosa cells of preovulatory follicles^31^, was not altered by hCG in Ganirelix-treated females supplemented or not by eCG (Supplementary Fig. 2A). LHCGR signaling was efficient, however, in increasing both the expression of thecal-specific steroidogenic factors and testosterone levels (Supplementary Fig. 2B and C). Overall, these findings suggest that the high FSH levels of the infantile period stimulate granulosa cell differentiation to some extent, but not sufficiently for the follicle to reach the terminal stage of maturation.

## Discussion

Mini-puberty of infancy, which is characterized by the postnatal activation of the hypothalamo-pituitary ovarian (HPO) axis, is a phenomenon common to mouse, rat, monkey, sheep, cow and human^1^,^2^,^3^,^5^,^7^,^34^. The mechanisms underlying the postnatal gonadotropin increments remain unclear; it has been advanced, however, that this postnatal activation results from the abrupt withdrawal of maternal placental estrogens, which exert an inhibitory effect on the HPO axis^5^. What effect this hypergonadotropic environment has on early ovarian activity had not been clearly analyzed. Using qualitative and quantitative methods in addition to the most reliable approach to measure steroids, *i.e.*, GC-MS, this study is the first to thoroughly analyze the role of FSH in ovarian physiology during the infantile period. Importantly, it demonstrates that high FSH levels permit ovarian steroidogenesis without stimulating follicular growth and allowing terminal follicular maturation. We discovered that it involves a regulatory mechanism whereby high FSH concentrations do not similarly impact the expression or activity of its targets involved in steroid production, follicular growth and maturation.

Our data emphasize previous observations that gonadotropins are already involved in the regulation of ovarian steroidogenesis in postnatal life, prior ‘true’ puberty^14^,^24^,^35^. Besides showing a concomitant increase in pituitary gonadotrope activity and ovarian steroidogenesis, we provide experimental evidence *in vitro* and *in vivo* that FSH is responsible for this process. Indeed, high levels of FSH are required to induce *Cyp19a1* in granulosa cells of preantral/early follicles from the first follicular waves. The observation of aromatase expression in relatively small GFs in human infant ovaries when FSH levels are elevated suggests that postnatal activation of the pituitary also enhances ovarian steroidogenesis through relatively small GFs in humans. Several studies have suggested that infantile GFs differ from those in adults, their granulosa cells arising from a population of somatic cell precursors that differentiate earlier during fetal life; besides, infantile GFs come from a population of follicles that start growing as soon as they form, at least in rodents^27^,^36^,^37^,^38^. Given the large number of studies using the infantile ovary as a relevant model of adult ovarian physiology, addressing whether this early FSH responsiveness is unique to infantile GFs or if it could also occur in size-matched follicles in the adult ovary needs to be clarified.

Despite the premature endocrine maturation of GFs and elevated gonadotropin levels, no terminal follicular maturation and no ovulation occur during the infantile period^21^,^26^. In the present report, we bring experimental evidence that despite its tremendous increase, FSH does not drive the sequence of events required to achieve these processes, that is follicular growth where granulosa cells proliferate and the antral cavity develops, and then follicular maturation wherein mural granulosa cells become LH-responsive and activate EGFR signaling to trigger ovulation. Our study does not question the fact that FSH is required for basal follicular growth up to the preantral/early antral stage during the infantile period, as shown previously^17^,^18^,^19^,^20^. But it supports the idea that elevated concentrations of FSH are unable to support subsequent growth of those follicles. Indeed, the experimental decrease of the FSH peak does not impair their number and size, although it affects ovarian production of E2. Conversely, the addition of exogenous gonadotropins to infantile mice is inefficient in stimulating their growth, notwithstanding enhanced E2 production. This treatment becomes efficient in stimulating follicular growth in our mouse colony when circulating FSH has returned to moderate levels, after 21 dpn (data not shown). Our findings are consistent with observations showing that preantral/early antral follicles display a slower growth at 14 dpn than at older prepubertal ages in the mouse^26^, but in apparent contradiction with a study in mouse models of follicle tracing^37^. In addition, an inverse relationship has been observed between antral follicle count and FSH levels in ewe lambs during mini-puberty^39^. Our study may provide some cues to understand why the aberrant elevation of postnatal FSH concentrations for several months following birth in prematurely born infant girls is associated with enhanced E2 levels but with decreased number of antral follicles^8^,^40^. It also raises the intriguing possibility that elevated FSH levels in adult patients with premature ovarian failure might be detrimental for follicular growth^41^.

By investigating the mechanisms underlying the refractoriness of follicular growth to FSH, we discovered that at high levels FSH becomes unable to stimulate cyclin D2-dependent follicular growth. Indeed, our *in vitro* studies using an organotypic culture system indicate that FSH induces *Ccnd2* expression in infantile ovaries only if present at a low, and not at a high concentration in the medium. *In vivo*, lowering the FSH peak to 20% of its normal value by Ganirelix efficiently restores FSH-induced *Ccnd2* expression in the ovary. This is accompanied by an increase in granulosa cell proliferation as shown by higher numbers of phospho-histone H3 and BrdU-positive granulosa cells in preantral/early antral follicles; these two effects are abolished by co-treatment with eCG. The existence of such a regulation *in vivo* is also suggested by the fall in *Ccnd2* expression when circulating FSH rises during the infantile period. We also report here that high FSH concentrations stimulate LHCGR expression in granulosa cells of immature GFs. However, LHCGR signaling is not functional in granulosa cells at this stage, at least in regulating both *Cyp19a1* and *Ccnd2* expression. Thus, the absence of terminal maturation of GFs would not solely result from the lack of FSH-mediated follicular growth, but also from the inability of granulosa cells to fully differentiate in response to gonadotropin hormones at this stage.

**Figure 5:**
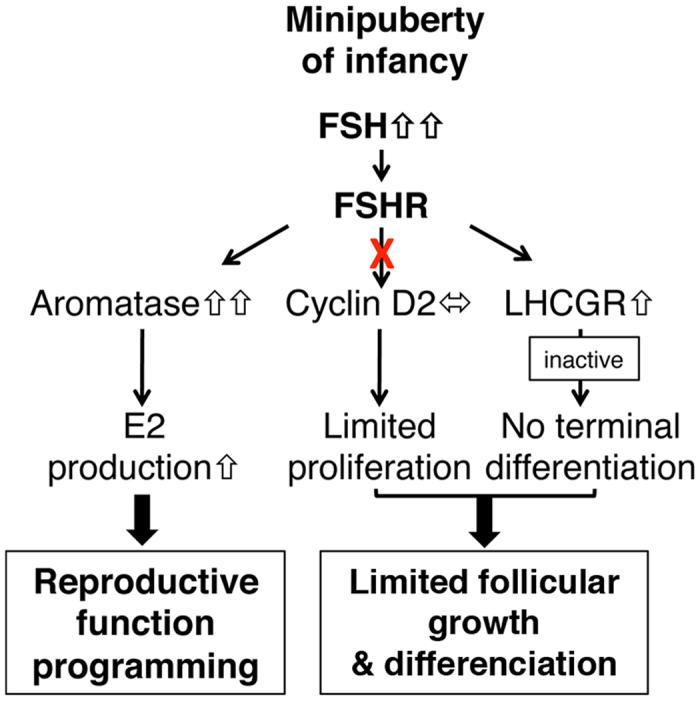
Proposed model of the effect of sustained postnatal FSH elevation on the infantile ovary. FSH increases the expression of *Cyp19a1* to activate E2 biosynthesis. However, at those high concentrations, FSH is unable to induce *Ccnd2* expression in granulosa cells, and thereby it loses its stimulatory action on follicular growth. In addition, FSH stimulates *Lhcgr* expression in granulosa cells but LH signaling pathway remains inactive in contrast with that in preovulatory follicles from cyclic females. Thus, during postnatal life, the dramatic elevation in FSH ensures the supply in E2 for reproductive function programming without inducing excessive follicular growth and premature follicular maturation.

The present report unveils the existence of decoupling in common regulatory pathways of FSH during the infantile period. This is well illustrated by the differential action of high FSH concentrations on the regulation of the expression of *Cyp19a1* (strongly induced) and *Ccnd2* (no induction) to trigger steroidogenesis while not supporting subsequent follicle growth. The fact that these events can proceed independently is further emphasized by previous studies showing that FSH treatment up-regulates the expression of *Cyp19a1* in the ovary of immature *Ccnd2*^*−/−*^ mice despite the absence of granulosa cell proliferation^31^. Since FSH signals through different pathways in addition to that of protein kinase A to selectively regulate target genes in granulosa cells^42^,^43^, we hypothesize that high FSH concentrations may stimulate downstream pathways involved in steroidogenesis while shutting down those involved in cell proliferation. Since the oocyte plays a major role in the development of ovarian follicles through the secretion of paracrine factors that regulate proliferation, steroidogenic ability and differentiation of granulosa cells^44^,^45^, it would be of interest to determine the possible contribution of the oocyte in the function of the infantile ovary. Overall, our findings establish that elevated FSH levels during the postnatal period essentially induce substantial ovarian endocrine activity by stimulating steroidogenesis with a minimal impact on other processes (Fig. 5). Such a mechanism would ensure E2 supply for the subsequent establishment of reproductive function while preserving the ovary from premature follicular maturation.

## Methods

### Animals

All studies were conducted on C57BL/6JRj mice aged 7 to 21 dpn and in adult cycling females that were born at the animal facility from genitors purchased at Janvier Labs (Le Genest St Isle, France). Mice were maintained under controlled conditions (12 h light/dark cycle) with food (Scientific Animal Food and Engineering (SAFE), A03–10) and water available *ad libitum*. The day of birth was designed as 0 dpn. Vaginal cells from females aged 4 to 6 months were collected by daily saline washes and analyzed after hematoxylin and eosin staining. Stages of the estrous cycle were characterized by predominant nucleated cells, predominant cornified epithelial cells and predominant leukocytes for proestrus (PE), estrus (E) and diestrus 2 (D) stages, respectively. Mice were anesthetized with a mix of ketamine (Imalgene^®^ 1000) and xylazine (Rompun^®^ 2%) to collect the blood by cardiac puncture. Blood was allowed to clot at room temperature for at least 15 minutes, and then centrifuged at 5000 g for 5 minutes to obtain the serum. After cervical dislocation, ovaries were collected, frozen in liquid nitrogen and stored at −80 °C for RNA and protein extraction, or fixed in 4% paraformaldehyde (PFA) for *in situ* hybridization, or immediately used for organotypic cultures. Experiments were performed in accordance with the ethics guidelines of the French Ministry of Agriculture and were approved by the ethics committee of University Paris 7.

### Animal treatments

Ten μg of Ganirelix acetate (Orgalutran^®^, N.V. Organon, Puteaux, France) or saline was subcutaneously injected twice on prepubertal female mice, at 12 and 13 dpn. In addition to Ganirelix, 13 dpn mice received an intraperitoneal injection of either 5 IU of human Chorionic Gonadotropin (hCG) (N.V. Organon), or 5 IU of equine Chorionic gonadotropin (eCG) (N.V.Organon), or saline. Injections (less than 12.5 μl) were performed with Hamilton syringes connected with catheters and needles (Phymep, Paris, France). Mice were then dissected at 14 dpn to collect blood and ovaries. For the superovulation procedure, mice received 5 IU of equine chorionic gonadotropin (eCG) followed 48 hours later by a 5-IU injection of hCG. Females were killed 20 hours later. Their oviducts were retrieved and placed in hyaluronidase in order to visualize any oocyte removed from the ampulla. For BrdU incorporation studies, females received a single injection of 100 mg/kg BrdU the day before dissection.

### Determination of serum estradiol levels

Gas chromatography coupled with mass spectrometry (GC-MS) procedure was used to determine the levels of E2 in the serum, as described previously^46^. After clotting, sera were kept at −80 °C until hormone assays. Randomized serum samples from two to three prepubertal animals were pooled. E2 was assayed in two steps. Additional information can be found in *SI Materials and Methods* and Table S1.

### Gonadotropin measurements

LH and FSH were simultaneously assayed in 10 μl of serum simplicate from the same serum samples, using the Luminex technology with the rat pituitary magnetic bead panel Milliplex Map kit (Merck-Millipore, Nottingham, UK) in accordance with the manufacturer’s instructions. Samples were run on a Bioplex-200 instrument (Bio-Rad, Marnes-La-Coquette, France) and concentrations were calculated using a five parameter logistic fit curve (5PL) generated from the standards by the Bio-Plex Manager 6.1 software (Bio-Rad, Marnes-La-Coquette, France). The sensitivity of the assays was 32 pg/ml for FSH and 3.2 pg/ml for LH. The inter-assay coefficient of variation was 5.4% for FSH and 3.2% for LH. The intra-assay coefficient of variation was 7.6% for FSH and 5.9% for LH.

### Cultures of postnatal ovaries

Ovaries from 12 dpn female mice were placed on cell culture inserts (Millicell #PICM01250, Millipore, Guyancourt, France) on the top of 400 μl of culture media containing RPMI without red phenol, fetuin (Sigma, #F2379), insulin (Sigma, #I0516), transferrin (Sigma, #T8158), sodium selenite (Sigma, #S5261) and BSA (Euromedex, Souffelweyersheim, France) in 24-well plates for 24 hours. Then, the culture medium was replaced with 400 μl of fresh medium containing purified ovine pituitary FSH (50, 200 or 500 ng/ml) provided by Dr R. Counis (University Paris-Diderot, France). After 8 hours of gonadotropin treatment, ovaries were snap-frozen in liquid nitrogen and stored at −80 °C for RNA extraction.

### RNA extraction, reverse transcription and quantitative real-time PCR

Single frozen ovaries were lysed with TissueLyser (Qiagen, Courtaboeuf, France) in RLT buffer from RNeasy mini kit (#74106, Qiagen), following manufacturer’s protocol. The extraction of total RNA was performed on columns and eluted with 30 μl of sterile water. The concentration and the quality of RNAs were assessed by the ratio of 260/230 nm and 260/280 nm with the Nanodrop device (Thermo Scientific, France). Total RNA (100 to 200 ng) was reverse transcribed with Superscript II (Invitrogen, Cergy Pontoise, France) and random primers (Invitrogen) following manufacturer’s instructions. Primers used for quantitative real-time PCR are listed in Table S2. *Hprt* was used as a reference gene to normalize the data. Real-time PCR was performed with Lightcycler^®^ 480 SYBR Green I Master (Roche Molecular Biochemicals, La Rochelle, France) and Light Cycler instrument (Roche) as previously described^47^.

### *In situ* hybridization

Ovaries were collected and fixed for 1–2 hours in 4% PFA, rinsed in PBS and placed in 18% sucrose before being embedded in Optimal Cutting Temperature (OCT) (Cellpath, Newtown, UK). *In situ* hybridization was performed on frozen sections with digoxigenin-11-UTP labelled RNA probes, as previously described^25^,^47^.

### Tissue processing and immunohistochemistry

For histological examination of mouse ovarian morphology and follicle counts, ovaries were fixed in 4% paraformaldehyde for at least 1 h, dehydrated in alcohol, and paraffin-embedded using standard protocols. Sections of 7-μm thickness were mounted on glass slides and stained with hematoxylin and eosin. Only follicles at the preantral/early antral stage (several layers of granulosa cells and diffuse or small antral cavities) located in the central part of the ovary were considered. Their number and surface was analyzed in every fifth histological section by ImageJ (ImageJ 1.64; Wayne Rasband, NIH). Immunostaining of Serine 10 phosphorylated histone H3 (Cell Signaling Technology, #9701) and BrdU (#ab152095, Abcam) was performed as described^48^,^49^. Counting of BrdU and P-HH3-positive granulosa cells in preantral/early antral follicles was performed using ImageJ. Immunostaining for fibronectin (Sigma, #086K4803) and cyclin D2 (Santa-Cruz biotechnologies, sc-593) was performed on frozen mouse ovarian sections, as previously reported^47^.

Formalin-fixed paraffin-embedded ovaries from girls aged from 2 to 6 postnatal months dying suddenly and unexpectedly in the hospital-university of Rennes were accrued under the French autopsy law that allows the use of such tissues for in-depth anatomopathological examination (Law 94–654 published on 29 July 1994). Autopsy was performed with informed consent of the parents in all cases. Adult human ovaries were provided by the Laboratory of Pathology of Gustave Roussy Institute (Villejuif, France) following prophylactic removal due to breast cancer between 1975 and 1980 with informed consent was obtained by the patients. These methods were carried out in accordance with relevant guidelines and regulations. All the experiments and experimental protocols on human subjects were approved by the institutional committee of the French agency for biomedical research (Agence de la Biomédecine, Saint-Denis la Plaine, France). Adult ovaries did not present any pathological aspect, showing corpora lutea at various stages of regression compatible with at least three successive ovulatory cycles. Immunohistochemistry was performed as previously described^48^. Slides were incubated overnight with primary antibodies for AMH (generously provided by Dr JY Picard) and aromatase (Sigma, #A7981). They were counterstained in hematoxylin, dehydrated and mounted in Permount (Fisher Scientific).

### Western blot

Pooled (n = 2 ovaries at 7 dpn) or single frozen ovaries were lysed with TissueLyser (Qiagen) in Frackelton buffer (10 mM Tris HCl, 50 mM NaCl, 1% Triton, 30 mM NaPPi, 50 mM NaF, 5 μM ZnCl_2_, 100 μM Na_3_VO_4_, 1 mM DTT, 1X protease inhibitor (Roche), 1X phosphatase inhibitor [Thermo Scientific]). After a 30-minute incubation on ice, lysates were centrifuged at maximal speed for 30 minutes, and supernatants were collected. Thirty μg of lysates were used for the determination of aromatase (Sigma, #A7981), cyclin D2 (Santa-Cruz, #sc-181) and GAPDH (Santa-Cruz, #sc-25778). Membranes were incubated in 10% milk for 60 minutes and incubated overnight with primary antibodies at 4 °C. The following day, the membrane was incubated with an anti-rabbit HRP-linked IgG antibody (dilution: 1/3000) for 2 h. Proteins were detected by chemiluminescence with ECL using CCD camera (LAS 4000, FujiFilm).

### Statistical analyses

All data were analyzed by Prism 6 (version 6.0, GraphPad Software). Statistical analyses were performed with the use of Student t-test or one-way ANOVA depending on the experimental setting. Data are shown as means ± SEM. A *P* value < 0.05 was considered as significant.

## Additional Information

**How to cite this article**: François, C. M. *et al*. A novel action of follicle-stimulating hormone in the ovary promotes estradiol production without inducing excessive follicular growth before puberty. *Sci. Rep.* **7**, 46222; doi: 10.1038/srep46222 (2017).

**Publisher's note:** Springer Nature remains neutral with regard to jurisdictional claims in published maps and institutional affiliations.

## Supplementary Material

Supplementary Information
