# Supplementary material for: A novel action of follicle-stimulating hormone in the ovary promotes estradiol production without inducing excessive follicular growth before puberty
Source: Sci Rep. 2017 Apr 11;7:46222. doi: 10.1038/srep46222 (PMC5387682; doi:10.1038/srep46222)

Supporting Information

A novel action of follicle-stimulating hormone in the ovary promotes estradiol production without inducing excessive follicular growth before puberty

Authors: Charlotte M François, Florence Petit, Frank Giton, Alain Gougeon, Célia Ravel, Solange Magre, Joëlle Cohen-Tannoudji, Céline J Guigon

SI Materials and methods

Determination of testosterone and estradiol levels in serum

Gas chromatography coupled with mass spectrometry (GC-MS) procedure was used to determine the levels of sex steroids in the serum, as described previously (Giton et al, 2015). After clotting, sera were kept at -80°C until hormone assays. Randomized serum samples from two to three prepubertal animals were pooled. Testosterone (T) and estradiol (E2) were assayed in two steps by GC/MS. Briefly, sera were overloaded with deuterated steroid internal standards (CDN isotopes Inc, Canada), and extracted with 1-chlorobutane. The organic phases were purified on conditioned silica mini-columns. T and E2 were derivatized with pentafluorobenzoyl chloride (103772-1G, Aldrich, Steinheim, Germany). The final extracts were reconstituted in isooctane and transferred into conical vial for injection into the GC system (6890N, Agilent Technologies, Massy, France) by using a 50% phenylmethylpolysiloxane VF-17MS capillary columns (20 m x 0.15 mm, internal diameter, 0.15 µm film thickness) (Varian, Les Ulis, France). A HP5973 (Agilent Technologies, Massy, France) quadrupole mass spectrometer equipped with a chemical ionization source and operating in single ion monitoring (SIM) mode was used for detection. The reagent gas for the negative chemical ionization mass spectrometry detection (NCI) was methane. The GC was performed in the pulsed splitless mode with a 0.5-min pulsed splitless-time. The temperature in the oven was initially 150°C for 0.5 min, further increased to 306°C at 22°C/min and held at 306°C for 3.6 min, and then to 334°C at 22°C/min and held at 334°C for 1.9 min. The injection port and transfer line temperatures were respectively 310 and 300°C. The flow-rate of helium (carrier gas) was maintained constant at 0.7 ml/min. The mass spectrometer CI source and quadrupole temperatures were respectively 200°C and 110°C. The linearity of steroid measurement was confirmed by plotting the ratio of the steroid peak response / internal standard (IS) peak response to the concentration of steroid for each calibration standard. Accuracy, target ions, corresponding deuterated internal control, range of detection, low limit of quantification (LLOQ), and intra & inter assay CVs of the quality control sera are reported in Table S1.

Table S1: GC-MS serum analytical control validation.

Accuracy	Analytes	Target ions analyte / IS (amu)	Range of detection (pg/ml)	Mean (43 runs) *Intra- & Inter assay CVs (%)*			
				LLOQ Mean (pg/ml) Intra- & Inter assay CVs (%)	Low QC Mean (pg/ml) Intra- & Inter assay CVs (%)	Middle QC Mean (pg/ml) Intra- & Inter assay CVs (%)	High QC Mean (pg/ml) Intra- & Inter assay CVs (%)
Accuracy for T and E2 measured by GC-MS -3.8 % - +3.5 % (n = 6)	T	T / T-d3 482 / 485	20 - 8100	20.1	485	2472	5945
				9.4 - 15.5	*2.2 - 3.1*	*2.1 - 2.6*	*1.2 - 2.1*
	E2	E2 / E2-d4 660 / 664	1.5 - 405	1.6	9.8	48.1	198.5
				10.3 - 16.2	*3.4 - 4.2*	*2.5 - 3.0*	*1.5 - 1.9*

Table S2: Primers used for qPCR.

Gene	Forward	Reverse	Size (bp)
Star	GTCATCAGAGCTGAACACGG	TGGCGAACTCTATCTGGGTC	163
Cyp11a1	GCACTTTGGAGTCAGTTTACATC	AGGACGATTCGGTCTTTCTT	180
Cyp17a1	CTTTCCTGGTGCACAATCCT	TACGCAGCACTTCTCGGATA	141
Cyp19a1	TACTTCATGTTACTTCTCGTCGC	TATCCTCGATCTTTATGTCTCTGTCAC	103
Hsd3b1	CAGCCAGGGGCCTTCGAGAC	GCTGGCATTAGGGCGGAGCC	143
Hsd17b1	GCAGTGCACACAGCCTTCTA	GCTCAGGGCTTGCTCATAAC	123
Ccnd2	CAGAAGGACATCCAGCCGTAC	TCGGGACTCCAGCCAAGAA	136
Lhcgr	ACCAAAAGCTGAGGCTGAGA	GGAGGCAGATGCTGACTTTC	123
Fshr	CTGGCATTCTTGGGCTCG	GGGCGGAATCTCGGTCA	101
Hprt	AGGACCTCTCGAAGTGT	ATTCAAATCCCTGAAGTATCAT	111

Supplementary Figure Legends

Fig. S1

The intra-ovarian expression of factors involved in sex steroidogenesis markedly increases during the infantile period. (*A*) The levels of *Lhcgr* and *Fshr* were determined during the infantile period (7-21 days postnatal) and in cyclic females at proestrus (PE), estrus (E) and diestrus 2 (D) in the mouse. Data were obtained by RT-qPCR and normalized to the mRNA levels of *Hprt* from 5 to 7 ovaries/group. (*B*) Schematic representation of the sex steroid biosynthesis pathway in large antral follicles. (*C*) The levels of *Star*, *Cyp17a1*, *Hsd3b1*, *Hsd17b1*, *Cyp11a1* and *Cyp19a1* were determined during the infantile period (7-21 days postnatal) and in cyclic females at proestrus (PE), estrus (E) and diestrus 2 (D) in the mouse. Error bars represent SEM. Different letters indicate significant differences ($P < 0.05$) between ages during the infantile period, as analyzed by one-way ANOVA. Statistical difference by

Student-*t* test of * *P* < 0.05, ** P < 0.01, *** P < 0.001 between 14 dpn females and a given stage of the estrous cycle. NS, not significant. (*D*) Determination of the abundance of P450 SCC (*Cyp11a1*) and aromatase (*Cyp19a1*) during the infantile period (7-21 days postnatal) and in cyclic females at diestrus 2 (D) and estrus (E) in the mouse. Data were obtained by Western blot, with GAPDH as a loading control. A representative immunoblot is shown.

Fig. S2

Activation of LH signalling by hCG has no effect on Cyclin D2 expression but it promotes the expression of P450 SCC and testosterone production in infantile ovaries. (*A*, *B*) The relative abundance of both *Ccnd2* and *Cyp11a1* (P450 SCC) mRNAs was determined by RT-qPCR and normalized to the mRNA levels of *Hprt* in females treated with Ganirelix (+), Ganirelix plus hCG (hCG), Ganirelix plus eCG (eCG), or Ganirelix plus hCG and eCG (hCG/eCG) (7-9 ovaries/group). (B) Circulating testosterone levels in 5-6 pooled serum in females treated with Ganirelix (+),Ganirelix plus hCG (hCG), Ganirelix plus eCG (eCG), or Ganirelix plus hCG and eCG (hCG/eCG). Error bars represent SEM. Different letters indicate significant differences (*P* < 0.05) between groups, as analyzed by one-way ANOVA.

Supplementary Figure 1

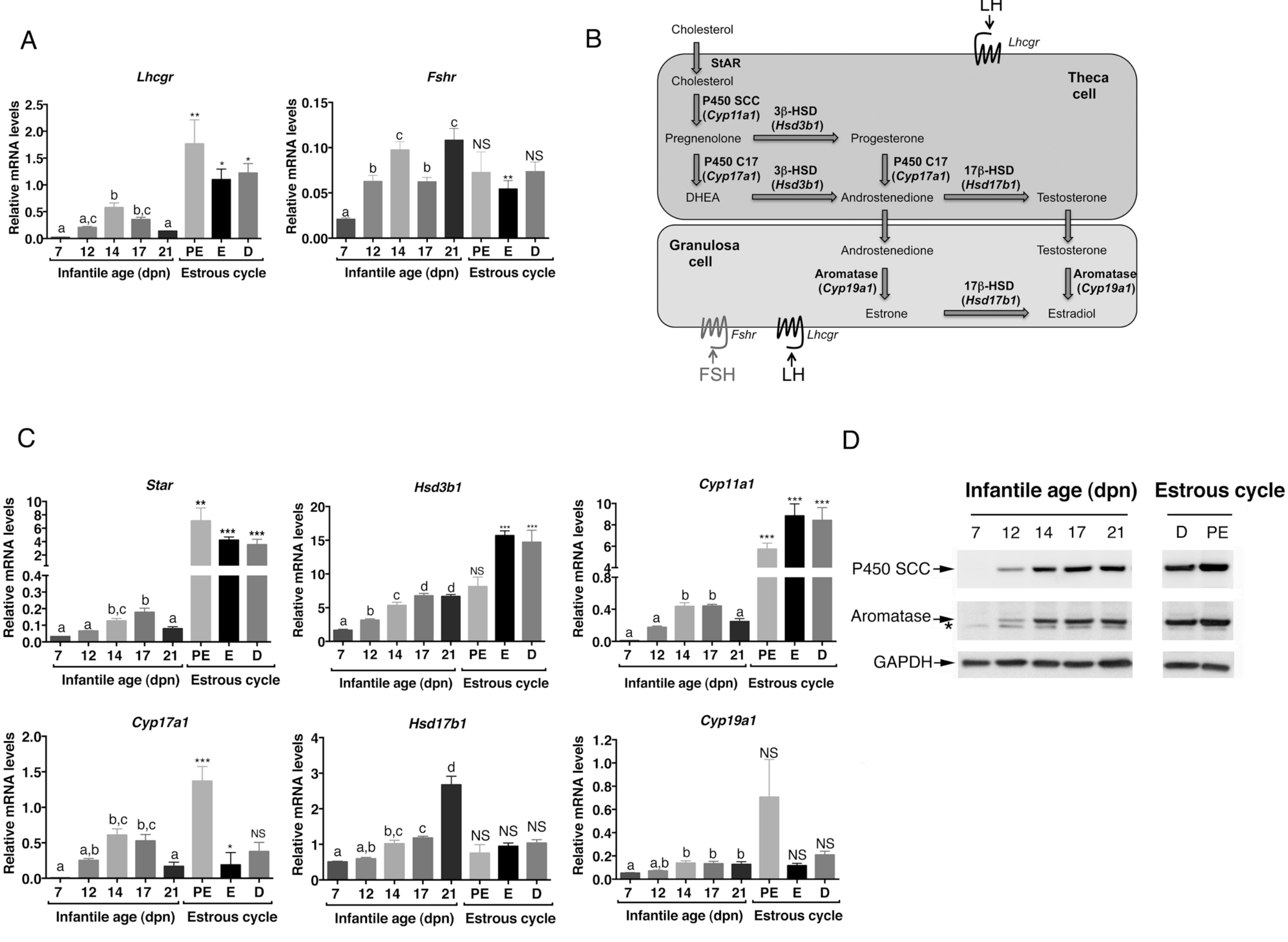

Supplementary Figure 2

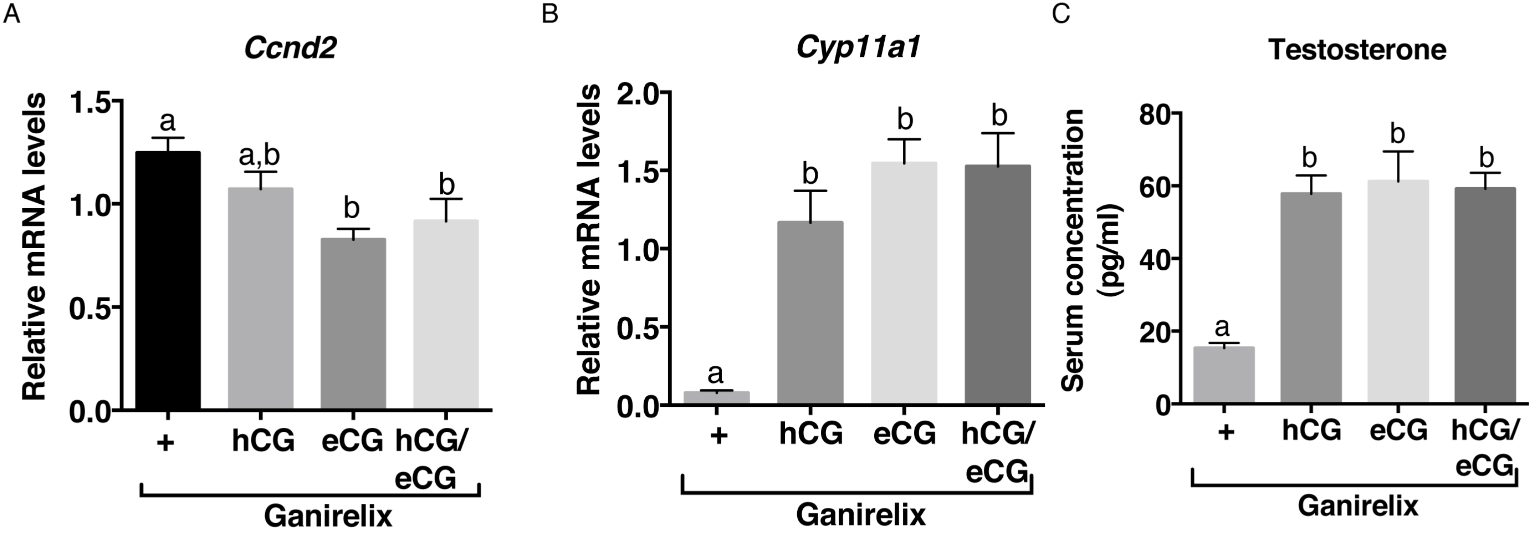

Supplement: Supplementary Information [file srep46222-s1.pdf]
